# Characterization of human adenovirus 35 and derivation of complex vectors

**DOI:** 10.1186/1743-422X-7-276

**Published:** 2010-10-19

**Authors:** Duncan McVey, Mohammed Zuber, Damodar EttyReddy, Christopher D Reiter, Douglas E Brough, Gary J Nabel, C Richter King, Jason G D Gall

**Affiliations:** 1 Department of Research, GenVec, Inc. 65 West Watkins Mill Road, Gaithersburg, MD 20874 USA; 2 Vaccine Research Center, NIAID, National Institutes of Health, Bethesda, MD 20892 USA; 3 Current Address: International Aids Vaccine Initiative, Brooklyn Army Terminal, 140 58th Street, Bldg A, Suite 8J, Brooklyn, NY 11220 USA

## Abstract

**Background:**

Replication-deficient recombinant adenoviral vectors based on human serotype 35 (Ad35) are desirable due to the relatively low prevalence of neutralizing antibodies in the human population. The structure of the viral genome and life cycle of Ad35 differs from the better characterized Ad5 and these differences require differences in the strategies for the generation of vectors for gene delivery.

**Results:**

Sequences essential for E1 and E4 function were identified and removed and the effects of the deletions on viral gene transcription were determined. In addition, the non-essential E3 region was deleted from rAd35 vectors and a sequence was found that did not have an effect on viability but reduced viral fitness. The packaging capacity of rAd35 was dependent on pIX and vectors were generated with stable genome sizes of up to 104% of the wild type genome size. These data were used to make an E1-, E3-, E4-deleted rAd35 vector. This rAd35 vector with multiple gene deletions has the advantages of multiple blocks to viral replication (i.e., E1 and E4 deletions) and a transgene packaging capacity of 7.6 Kb, comparable to rAd5 vectors.

**Conclusions:**

The results reported here allow the generation of larger capacity rAd35 vectors and will guide the derivation of adenovirus vectors from other serotypes.

## Introduction

Recombinant adenovirus (rAd)-based gene transfer vectors are currently under investigation in a variety of gene therapy and vaccine clinical trials. There are more than 370 such clinical trials that are ongoing for broad applications, including infectious diseases and cancer therapy http://www.wiley.com//legacy/wileychi/genmed/clinical/. Many of these trials are of recombinant adenovirus (rAd) vectors based on the human serotype 5 (Ad5) yet there are advantages to rAd vectors derived from other serotypes. The human adenoviruses have previously been shown to have different prevalence in populations around the world [1]. Exposure of human populations to adenovirus serotype 35 (Ad35) has been shown to be relatively rare based on the prevalence of Ad35 neutralizing antibodies in sera [1-5]. Because neutralizing antibody could interfere with the efficacy of viral gene transfer vectors, the low seroprevalence of Ad35 makes it an attractive candidate for derivation of viral vectors [1,4,6,7].

The Ad35 genome has an overall organization similar to all adenoviruses [1,6,8], which facilitated the derivation of E1-deleted rAd35 vectors. However, the reported Ad35 genome annotations were based on sequence homology analyses without experimental evidence and some of the initial rAd35 vectors were found to have genetic instability due to the inadvertent deletion of the promoter for the structural protein IX (pIX) [9]. Thus, homology analysis was limited in predicting functional regions of the Ad35 genome. To develop a complex rAd35 vector with up to three large deletions we attempted to characterize the Ad35 life cycle relevant to rAd35 viral vector productivity, stability, and capacity for foreign DNA. Essential sequences were identified in E1 and E4, the sequences were deleted, and the effects of the deletions on viral gene transcription were determined. The non-essential E3 region was also deleted from rAd35 vectors and a sequence was found that unexpectedly affected late fiber gene transcription, with subsequent effects on viral fitness. The packaging capacity of rAd35 was dependent on pIX and viral capsids with pIX packaged viral genomes up to 104% of the wild type genome size, whereas pIX-deficient capsids had a packaging limit of less than 100% wild type genome size. These data were used to make an E1-, E3-, E4-deleted rAd35 vector. This rAd35 vector with multiple gene deletions has the advantages over previous rAd35 vectors of a second block to viral replication (i.e., E1 and E4 deletions) and an expanded transgene packaging capacity totaling 7.6 Kb, comparable to rAd5 vectors.

## Results

### Ad35 capsid components and identification of the early/late switch

**Figure 1:**
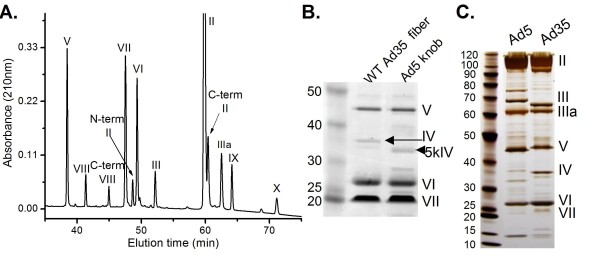
**Identification of Ad35 viral particle proteins**. **(A) **Representative reverse-phase HPLC chromatogram of Ad35 capsid proteins with identities shown. N-term II, C-term II = amino- and carboxy-terminal portions, respectively, of protein II. **(B) **Ad35 SDS-PAGE protein IV (fiber) identification. Ad35 viruses with wild type (WT) fiber or a genetically modified fiber protein containing the Ad5 fiber knob (5kIV) with predicted molecular masses of 35.4 and 33.9 kDa are indicated by an arrow and arrow head, respectively. Proteins were analyzed by SDS-PAGE and visualized by Deep Purple stain. Molecular weight markers are in the first lane along with their molecular weight in kDa. **(C) **SDS-PAGE protein assignments for Ad5 and Ad35 capsid proteins stained by silver. Purified virus was loaded at 2.5 × 10^10 ^particles per lane. Molecular weight markers are in the first lane along with their molecular weight in kDa. Ad35 capsid proteins are annotated to the right.

To facilitate the derivation of rAd35 vectors with multiple genome deletions the protein components of wild type virions were determined. Twelve significant protein peaks were identified by reverse-phase HPLC (rp-HPLC) of purified, denatured viral particles (Figure 1A). Effluent fractions from rp-HPLC were analyzed for protein molecular weights by SDS-PAGE and mass spectroscopy (Table 1). The combination of whole protein or tryptic peptide molecular weight data determined by mass spectroscopy was used in conjunction with the apparent molecular weights by SDS-PAGE to assign identities to all of the rp-HPLC peaks and many SDS-PAGE bands. However, the fiber protein (protein IV) was not identified by rp-HPLC. The identity of protein IV in SDS-PAGE was determined by comparing two Ad35 viruses that differed only in the fiber protein. The wild type Ad35 fiber protein was predicted to have a molecular weight of 35.4 kDa while the mutant protein (5kIV), in which the fiber knob was replaced with that from Ad5, was 33.9 kDa (Figure 1B). Taken together, these data allowed the assignment of identities for ten proteins in rp-HPLC chromatograms and for seven proteins in SDS-PAGE analysis (Table 1 & Figure 1C).

**Table 1:** Identification of Ad35 viral particle proteins.

	Protein mass spectroscopy molecular weight (MW) determination*					
			
	**Whole protein MW comparison**		**Tryptic peptide Identification**		SDS-PAGE molecular weight**	
			
**Protein**	**Experimental, theoretical (Da)**	**Δ (ppm)**	**No. of peptides**	**p-value**	**Identification**	**Apparent mass**
						
II (Hexon)	107119.25, 107251.20	12.3	n.d.	N/A	Size and abundance	107 kDa
II, n-term. fragment	n.d.	N/A	4	8.50 × 10^-13^	Tryptic peptide fingerprint	16 kDa
II, c-term. fragment	n.d.	N/A	n.d.	N/A	Abundance in RP--HPLC	90 kDa
III (Penton Base)	n.d., 62916.92	N/A	7	6.30 × 10^-6^	SDS-PAGE of RP-HPLC fraction	64.9 kDa
IIIa	64000.63, 63942.38	9.1	10	2.10 × 10^-9^	SDS-PAGE of RP-HPLC fraction	61 kDa
IV (Fiber)	n.d., 35351.52	N/A	n.d.	N/A	Size shift of modified protein	35.3 kDa
V	40033.65, 40099.01	16.3	5	2.10 × 10^-6^	Inference from MW	46 kDa
VI	21783.45, 21728.76	25.2	n.d	N/A	SDS-PAGE of RP-HPLC fraction	28 kDa
VII	18784.53, 18738.47	24.6	3	Manual analysis	Inference from MW, silver and Deep Purple staining intensity	23.8 kDa
VIII	12204.10, 12190.81	10.9	5	1.50 × 10^-9^	Inference from MW	12 kDa
VIII, c-term	7644.21, 7697.31	69	n.d.	N/A	N/A	N/A
IX	14149.42, 14193.94	31.4	2	Manual analysis	Inference from MW and IX deficient virus	14.1 kDa

*Determined by analysis of rp-HPLC fractions

**Determined by analysis of rp-HPLC fractions and whole, denatured viral particles

ppm ^: ^parts per million

n.d.: not done

N/A: not applicable

**Figure 2:**
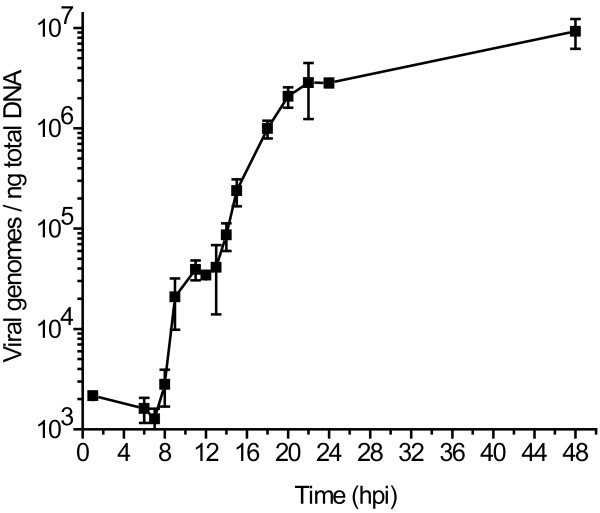
**Viral DNA synthesis**. A549 cells were infected with wt Ad35 at an MOI of 5 focus forming units (FFU) per cell, the viral genome number at each time point was determined by qPCR with primers and probe to pIX coding sequences, standardized to total DNA, and expressed as viral genomes per ng of total DNA. Triplicate infections were performed for each time point; standard deviation error bars shown; hpi = hours post infection.

Because adenovirus transcriptional patterns differ dramatically before and after viral genome replication [8], we determined the onset of viral DNA replication by QPCR. A549 cells were infected with wild type Ad35 and the number of copies of viral DNA determined at time points from 1 to 48 hours post-infection (hpi). The amount of viral DNA remained unchanged from 1 to 8 hpi and then increased, demonstrating that the onset of viral DNA replication was between 8 and 9 hpi (Figure 2). This information provided the basis to look at differences in viral gene expression between the early and late phases of viral infection. Taken together, these data allowed for subsequent identification of effects of deletions on both transcription and protein expression levels.

### Characterization of the Ad35 E1B/pIX region

An objective for optimal rAd vector design was to maximize the space available in the genome for insertion of transgene expression cassettes. All coordinates of viral locations are based on the Ad35 Holden sequence (GenBank accession number AY128640) To design the largest deletion of the E1 region we determined if E1B and pIX transcripts were generated from overlapping genome sequences that would be perturbed by deletion of E1B. First, DNA sequencing of a λ cDNA library from Ad35-infected cells identified the polyadenylation site and the junctions for two introns. The cDNA clones that included E1B and pIX sequences showed polyadenylation occurring at the position equivalent to nucleotide 3952. Thus, the Ad35 E1B and pIX transcripts utilized the polyadenylation hexanucleotide signal at nucleotides 3925-3930 and 3929-3934 inclusive. The left-most splice junction identified in the cDNA library joined base pairs 2180 and 3231, placing it in the open reading frame previously annotated to encode the E1b 494R protein which is analogous to Ad5 E1B 55k [6]. A second splice junction joined base pairs 3402 and 3480, placing it in the intergenic region between E1B and pIX coding sequences with the 3' splice junction (bp 3480) five nucleotides upstream of the pIX initiation codon. The proximity of the 3' splice junction implied that the promoter for pIX was located farther upstream than would have been predicted by homology to the well-characterized species C adenoviruses [10].

**Figure 3:**
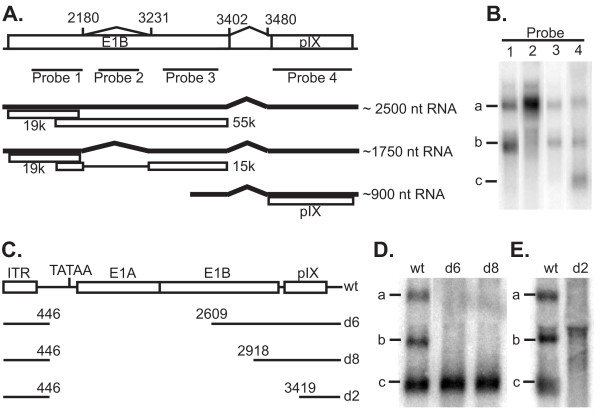
**E1B and pIX transcription mapping**. **(A) **Schematic of E1b and pIX sequences, splice junctions shown as ^ with their coordinates that were identified by cDNA analysis, and probe locations (not to scale). Base pair coordinates of the probes are: 1 = 1641-1903, 2 = 2527-3046, 3 = 3283-3359, 4 = 3511-3853. Solid thick lines depict transcripts a, b and c as determined from cDNA and northern blot analysis (panel B) with apparent sizes given to the right in nucleotides (nt). The predicted proteins (boxes) are labeled with the name of their Ad5 homologues pIX, E1b19K, E1b55K [6], and E1b15K [11]. **(B) **Northern blot analysis of pIX transcripts in cells infected with wild type Ad35. Probes 1, 2, 3, 4 from panel A; a, b, c, = RNAs corresponding to putative mRNAs. **(C) **Schematic of Ad35 E1 region deletions with Ad35 coordinates corresponding to deletion junctions shown above each line. The viral left ITR, E1A TATAA box, and E1A, E1B, and pIX coding sequences are represented. **(D) **and **(E) **Northern blot analysis of transcripts in cells infected with wild type Ad35 (wt) or Ad35 viruses with E1 deletions and hybridized to probe 4. Location of transcripts a, b and c are indicated to the left of each blot.

Based on the exon - intron junctions found by the cDNA library analysis and the conserved structure of human adenovirus E1/pIX regions, a set of probes for detection of RNA transcripts were designed to span the predicted introns and exons (Figure 3A). Northern blot analysis of steady-state RNA from wild type Ad35 infected cells detected three distinct transcripts, 'a', 'b', and 'c' (Figure 3B). A separate northern blot analysis with strand-specific probes demonstrated the three transcripts were generated from the E1/pIX coding strand (data not shown). The largest transcript, transcript 'a,' hybridized to all four probes, consistent with an mRNA encoding E1B 214R and 494R proteins, homologs for Ad5 E1B 19K and 55k proteins, respectively (previously annotated by sequence homology [6]). Transcript 'b' hybridized to probes 1, 3, and 4 but not 2, identifying it as the doubly spliced transcript found by cDNA analysis. Transcript 'b' would be predicted to encode for E1B 19K homolog and a modified form of 55K with a predicted molecular weight of 15K, from removal of an intron as described for Ad5 [11]. The smallest transcript, 'c,' hybridized to only probe 4. In addition, nucleotide 3367 was the 5'-most nucleotide identified with the cDNA library, consistent with the identification of transcript 'c' encoding only pIX. None of the transcripts hybridized to a probe to the second intron (data not shown) suggesting that all three transcripts were spliced in this intergenic region. Thus, two alternative spliced transcripts of E1B were identified and the pIX transcript was identified and shown to have a 5' untranslated region in E1B, as annotated in Figure 3A.

**Figure 4:**
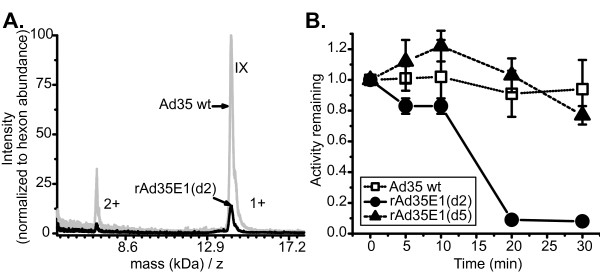
**Determination of pIX function**. **(A) **Relative abundance of pIX in viral particles. Approximately 1 × 10^11 ^particles of wild type Ad35 or Ad35 with the E1 deletion d2 were fractioned as in Figure 1A and collected for subsequent mass spectroscopy analysis. The analysis of the fraction corresponding to the peak denoted 'IX' in Figure 1A is shown here with the viral sources (Ad35 wt and rAd35E1(d2) indicated. The peak at 14.1 kDa/z is the singly charged protein pIX (1+) and the peak at 7 kDa/z is the doubly charged protein pIX (2+). The abundance of pIX was normalized to hexon abundance because the two proteins co-eluted. Based on intensity the rAd35E1(d2) virus contains ~10% of wild type pIX Ad35 levels. **(B) **Effect of pIX on heat stability of Ad35 virions. Virions were heat treated in triplicate at 48 C for the time indicated and activity determined by an FFU assay.

Recombinant Ad35 vectors with E1 region deletions and transgene expression cassettes were constructed (Figure 3C) and analyzed for E1B and pIX transcription. RNA from cells infected with the rAd35 vectors were analyzed by northern blot with the pIX gene probe (probe 4). Transcript 'c' was present in cells infected with two rAd35 vectors with the deletions d6 and d8, whereas the transcripts corresponding to E1B mRNAs were not detected (Figure 3D). In contrast, none of the transcripts were detected in cells infected with a rAd35 vector with the d2 E1 deletion (rAd35E1(d2)), even though the pIX gene was not deleted from the vector genome (Figure 3E). The RNA found between transcript 'a' and 'b' with the d2 deletion was not identified but could be a transcript that originated from the expression cassette found in the E1 region [12]. Based on the assignment of transcript 'c' as the pIX-encoding transcript, it was predicted that the d6 and d8 deleted rAd35 vectors would express pIX protein but rAd35E1(d2) would not. Although the pIX gene has been demonstrated as non-essential, the pIX protein effects the structure of the virus particle and its absence from the mature virion can increase the heat lability of the capsid and reduce the genome packaging limit, thus rAd35 vectors with the protein would have desired characteristics. The levels of pIX in the viral capsid were directly quantified by reverse phase HPLC and the level of pIX in rAd35E1(d2) virions was found to be ~10% of Ad35 wild type levels (Figure 4A). Because the d2 deletion was the relatively larger deletion size, it would provide for a larger capacity for foreign DNA. Thus, we determined whether the loss of pIX expression effected two functions of the viral capsid: viral particle integrity and capacity for packaging DNA. To restore pIX levels in the d2 genetic background, pIX was expressed from a heterologous promoter in the d2-deleted rAd35. The AAV P5 promoter was inserted immediately 5' of nucleotide 3419 in the d2 deletion backbone yielding deletion d5; the resultant rAd35 vector was designated rAd35E1(d5). Viral particle integrity was assessed by comparing the stability of virions after heat treatment. Wild type Ad35 and rAd35E1(d5) did not lose infectivity at 48°C while the infectivity of rAd35E1(d2) was reduced more than 100-fold in 20 minutes at 48°C (Figure 4B). These results are consistent with the pIX function of stabilizing the capsid and with a previous proposal for pIX in Ad35 [9]. The level of pIX in purified rAd35E1(d5) capsids was determined to be equivalent to those of wild type virus by rp-HPLC and SDS-PAGE (data not shown). Thus, loss of transcript 'c' correlated with loss of pIX incorporation into the virion such that pIX function was lost.

The packaging capacities of rAd35 vectors with deletions d2, d5, d6, and d8 were determined by assessing genetic stability. Genome rearrangements were detected by PCR analysis of the non-essential CMV expression cassette in the E1 region. Amplification products not present in the plasmid control reactions indicated rearrangements. A subset of amplification products were sequenced to confirm their E1 region origin and the nature of the rearrangement (data not shown). Viruses with the E1(d2) deletion showed E1 region rearrangements with genomes smaller than 100% of the wild type Ad35 genome size. A very clear transition occurred in stability of viral genomes between 97.2% and 99.3% of wild type genome sizes (Table 2). This transition was reproducible with all 6 viruses ranging in size from 99.3 to 100.4% showing genetic instability while the three viruses 97.2% and smaller were stable. In contrast, rAd35 vectors with the d5, d6, and d8 deletions, which express pIX, were genetically stable with genome sizes larger than the wild type genome size. The upper packaging limit of Ad35 appeared to be between 103.6% and 103.9% of wild type genome size. An Ad35E1(d6) virus with a genome size of 103.6% was stable through at least 5 passages, however Ad35(d8) viruses that were 103.9% and 104.1% started to show weak signs of genetic rearrangement in the sensitive PCR based packaging assay. This packaging limit is in good accord with the 105% limit that has been previously reported for Ad35 [13]. Taken together, the heat lability and genetic stability results directly demonstrate the importance of pIX protein in establishing the viral packaging capacity for Ad35.

**Table 2:** Ad35 viral vector packaging capacities.

**E1 deletion, transgene**	**Other genome modifications**	**pIX expression**	**Genome size (percent of wt)**	**Stability at Passage 3**
				
d2, no transgene	None	No	94.92	Stable
d2, green fluorescent protein (GFP)	None	No	96.96	Stable
Δ447 - 3326, GFP	None	No	97.22	Stable
d2, secretory alkaline phosphatase	None	No	99.33	Rearrangements
d2, luciferase	None	No	99.79	Rearrangements
d2, bacterial beta-glucuronidase	None	No	99.94	Rearrangements
d2, HIV Env gp140ΔCFIΔV1V2	None	No	99.95	Rearrangements
d2, HIV Env gp140ΔCFI	None	No	100.41	Rearrangements
d2, lacZ	None	No	103.68	Rearrangements
d8, eGFP	E3(HE), E4(dORF3-6)	Yes	85.60	Stable
d8, eGFP	E3(HE), E4(dAN)	Yes	87.30	Stable
d5^, HIV Env gp140ΔCFIΔV1V2	None	Yes	100.43	Stable
d8, HIV Env gp140ΔCFI	None	Yes	101.70	Stable
d6, Luciferase	None	Yes	103.60	Stable**
d8, HIV Env gp140ΔCFI	Fiber modified*	Yes	103.90	Rearrangements
d8, Luciferase	Fiber modified*	Yes	104.11	Rearrangements

^d2 with AAV P5 promoter for pIX expression

*Ad35 fiber shaft and knob replaced with Ad5 fiber shaft and simian Ad25 knob

** Through greater than 5 passages

The relationship of pIX expression to capsid integrity provided criteria for the selection of an optimal E1 deletion: maximum deletion of E1 sequences and retention of native control of pIX expression. The E1(d8) deletion was selected as the base for further rAd35 vector development. Recombinant Ad35E1(d8) vectors contained the largest E1 deletion tested that maintained wild type level of pIX transcription (transcript 'c'). In addition, the yield of rAd35E1(d8) viral progeny was consistently high, approximately 100,000 particles per cell when propagated on the 293-ORF6 cell line (data not shown).

### The Ad35 E4 Open Reading Frame 6 sequence was necessary and sufficient for E4 complementation

**Figure 5:**
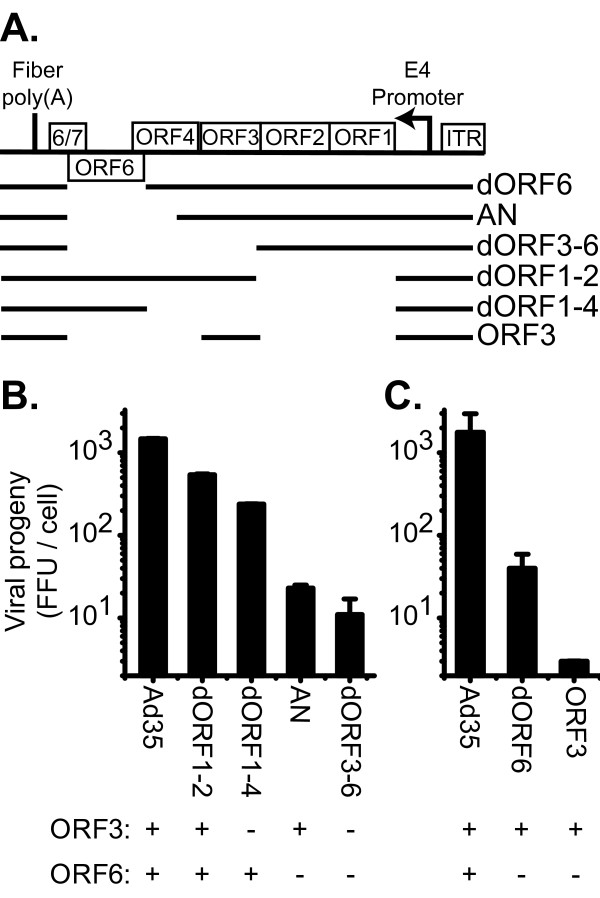
**Effect of E4 deletions on Ad35 growth**. **(A) **Schematic of Ad35 E4 region and deletions (not to scale). The previously identified open reading frames 125R, 145R, 117R, 122R and 299R, are identified by their corresponding Ad5 open reading frame (ORF) ORF1, ORF2, ORF3, ORF4 and ORF6 respectively [6]. The Ad35 ORF6/7 has not been previously described and has coordinates 32,978-32,805 + 32082-31830. The name of the deletion is given to the right with the solid lines indicating which E4 sequences are retained. The description of the junctions are as follows: dORF6 32,010-32,877 with the stop codon of ORF4 changed to TAG, AN 32,007-33,083 with sequence CTAGTCTAGACTAG inserted, dORF3-6 32,010-33,604 with ORF2 stop codon changed to TAG, dORF1-2 33,604-34,416, dORF1-4 32,974-34,416, and ORF3 32,007-33,254 with sequence GCGCGTCGCGA inserted followed by 33,607-34,416. **(B and C) **Generation of viral progeny on the PC3 cell line. Presence (+) or absence (-) of ORF3 and ORF6 coding sequences is noted below each rAd. Active viral particles were determined at 72 hpi.

Deletion of the E4 region would increase the space in the rAd35 vector genome for foreign DNA and provide a block to replication of the rAd35 genome in transduced cells. To facilitate the derivation of rAd35 vectors with multiple genome deletions, the sequences necessary for E4 function were mapped using Ad35 viruses with intact E1 regions. E1-wild type, E4-deleted rAd35 viruses (Figure 5A) were assessed for growth on PC3 cells, a human prostate cancer cell line without known E4-complementing activity. Viruses with E4 deletions of either ORF1 + ORF2 (dORF1-2) or ORF1 through ORF4 (dORF1-4) generated similar, although approximately 5-fold lower, viral progeny as wild type Ad35 (Figure 5B). In contrast, internal E4 region deletions of ORF4 through ORF6 (AN) and ORF3 through ORF6 (dORF3-6) generated approximately 100-fold less viral progeny, i.e., 10 to 20 infectious viral progeny produced per cell. These results indicated that deletion of ORF6 had the greatest effect on viral replication and ORF3 sequence was not sufficient for productive viral infection. In a second experiment, a virus with only ORF6 deleted showed an approximately 50-fold drop in viral progeny compared to wild type, and a virus with deletion of all open reading frames except ORF3 was also deficient in viral progeny production (Figure 5C). The ORF3 transcript was detected in cells infected with the ORF3 virus (data not shown), although determination of ORF3 protein expression was not attempted. Surprisingly, in contrast to other human serotypes, Ad35 ORF3 was not sufficient to provide E4 activity as demonstrated with the AN, dORF6, and ORF3 viruses which retained ORF3 sequence. Taken together, these data demonstrate that only ORF6 function must be absent for generating a replication deficiency.

### Transcription analysis of the Ad35 E4 region with deletions

**Figure 6:**
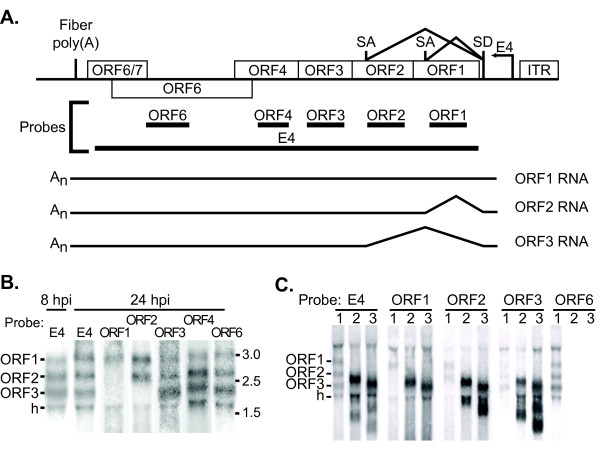
**E4 transcriptional analysis**. **(A) **E4 genomic and transcription map with probes used in northern blot analysis (not to scale). Genomic description is as in Figure 5 with the addition of putative splice donor (SD) and splice acceptor (SA) sites. The names of the identified RNAs are given to the right. ^ = splicing; A_n _= polyadenylation.. Base pair coordinates of probes: ORF1 = 34,045-34,412, ORF2 = 33,611-34,015, ORF3 = 33,258-33,611, ORF4 = 32.882-33,214, ORF6 = 32,012-32,887, E4 = 31,855-34,550. **(B) **Northern blot analysis of RNA from Ad35 wild type-infected 293-ORF6 cells at 8 and 24 hpi. Probes are indicated above each lane and predicted transcripts are labeled on the left of the gel. Numbers on the right denote migration of RNA size standards. **(C) **Northern analysis at 24 hpi of RNA from wild type Ad35 with the following E4 regions: 1 = wt; 2 = AN; 3 = dORF6. Labeled as in panel B. Identity of transcript h was not determined.

We next determined the transcriptional profile of the wild type Ad35 E4 region at early and late time points prior to deleting portions of the E4 transcription unit. A northern blot using a probe to the entire E4 region (E4 probe; Figure 6A) identified four and five individual transcripts at 8 and 24 hpi, respectively (Figure 6B). Because the five late transcripts appeared to include the four early transcripts and late phase RNA was more abundant, late phase RNA was used for further analysis. Open reading frame-specific probes identified three of the transcripts as having unique 5' open reading frames (Figure 6B). The ORF1 probe hybridized to only one transcript, implying that the ORF1 protein would be expressed from that transcript only. The ORF2 probe hybridized to the same transcript as the ORF1 probe and a second, smaller transcript, thus the ORF1 sequence was likely removed from the ORF2-encoding transcript. Similarly, the ORF3 probe hybridized to an even smaller RNA, suggesting that ORF1 and ORF2 sequences were absent and the ORF3 protein was expressed from this RNA. The ORF4 and ORF6 probes gave patterns indistinguishable from the entire E4 probe. The three predicted alternative spliced transcripts (ORF1, ORF2, and ORF3) could be aligned to the E4 region (annotated in Figure 6A). To determine whether the bands on the blots were E4 transcripts, we conducted northern blot analyses of RNA from cells infected with the E4-deleted rAd35 viruses dORF3-6 and AN. The transcripts identified by the probe for the entire E4 region decreased in size concomitantly with the E4 deletion in the virus (Figure 6C). A single transcript was detected with the ORF1 probe, two transcripts with the ORF2 probe and three transcripts with the ORF3 probe, while the ORF6 probe did not detect any transcripts in the E4-deleted rAd35 infected cells. These results confirmed the identity of the transcripts generated from the E4-deleted viruses as the ORF1, ORF2, and ORF3 transcripts. Interestingly, transcripts for neither ORF4 nor ORF6 were revealed in this analysis. It is possible their abundance was too low for detection by northern blot or the proteins are translated from one of the identified transcripts. The identity of the transcript labeled 'h' could not be determined (Figure 6B &6C). The ORF6 probe, which was not strand-specific, detected the transcript in cells infected with Ad35 with an intact E4 region but not in cells infected with rAd35 with ORF6 deleted (dORF3-6 and AN). However, deletion of these same sequences (dORF3-6 and AN) did not cause any change in the size of the h transcript when tested with the complete E4 probe. Thus, it was likely that the 'h' transcript was not generated from the Ad35 E4 region despite hybridizing to a probe of ORF6 sequences.

### Ad35 E3 region deletions and effects on fiber gene regulation

**Figure 7:**
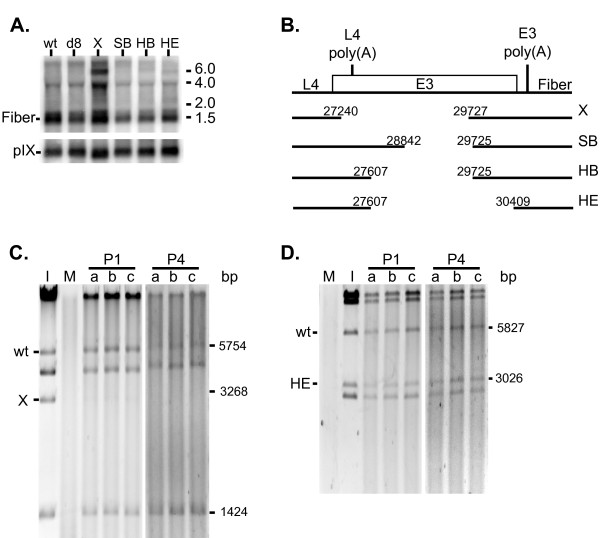
**Effect of E3 deletions on fiber transcription and virus fitness**. **(A) **Northern blot of 24 hpi total RNA from 293-ORF6 cells infected with wild type Ad35 (wt), E1-deleted rAd35 (d8), or rAd35 d8 constructs with the E3 deletions noted in panel B. The blot was hybridized with a fiber (top) or pIX probe (bottom); the fiber and pIX transcripts are labeled. Numbers on the right denote migration of RNA size standards (kilobases). **(B) **Schematic of the Ad35 E3 region and E3 deletions (not to scale). The coordinates of the nucleotides that form the deletion junctions are shown above the lines. L4 poly(A), E3 poly(A) = L4 and E3 polyadenylation hexanucleotide signals, respectively. **(C) **and **(D) **Viral vector growth competitions. rAd35 d8 E1-deleted vector was mixed with **(C) **rAd35 d8, E3(X)-deleted vector or with **(D) **rAd35 d8, E3(HE)-deleted vector. Relative change in genome amounts was determined by DNA restriction fragment analysis of the input mixture of viruses and each serial passage. DNA restriction fragment analysis uniquely identified each virus genome, which is indicated to the left of the gel and their fragment sizes in bp on the right. I = input mixed viruses used for initial infection; M = mock infected cells; P1 = initial infection; P4 = fourth passage; a, b, c = replicates. Restriction enzymes EcoRV and BlpI were used in panels C and D respectively.

For vectors derived from other serotypes, deletion of non-essential E3 sequences provided more space in the vector genome for foreign DNA and we sought to extend this strategy to Ad35. Due to the proximity and transcription orientations of the E3 and L5 regions, the effect of E3 deletions on fiber protein gene transcription, the sole product of L5 was determined. Sequence analysis of lambda cDNA library clones with fiber sequences identified a tripartite leader sequence with three splice junctions that joined nucleotides 5962 to 6981, 7052 to 9496, and 9582 to 30830. The identity of the leader sequence had previously been proposed based on bioinformatics [4]. The cDNA analysis also provided identification of two sites for polyadenylation addition for the fiber transcripts, corresponding to nucleotides 31,825 (uracil) and 31,831 (cytidine). The sizes of the cDNAs for fiber were approximately 1.5 kilobases (kb). Northern blot analysis with a fiber sequence probe of RNA from cells infected with wild type Ad35 identified at least two major transcripts, one at ~4.0 (kb) and one or more at ~1.5 kb (Figure 7A, lane "wt"). Strand-specific probes confirmed the transcripts to be encoded by the fiber sense strand of the viral genome (data not shown). Based on the sizes of the cDNAs, the 1.5 kb transcript was identified as encoding the fiber protein.

Once the fiber transcript was identified, the effects of E3 region deletions on fiber transcription were determined. A panel of E3 deletions was generated in the E1-deleted backbone rAd35(d8) (Figure 7B), and fiber transcripts were analyzed in cells infected with the E1- and E3-deleted rAd35 vectors. The E3(X) deletion appeared to increase the level of both the 4.0 kb and 1.5 kb fiber transcripts and a new RNA of approximately 6.0 kb was detected (Figure 7A). Phosphoimager quantification of the 1.5 kb and 4.0 kb bands showed a 2-3 fold increase relative to wild type Ad35 and rAd35(d8) (data not shown), while the level of the pIX transcript did not change. In contrast, E3 deletions SB, HB and HE did not affect the quantity or size of the fiber transcripts (Figure 7A). Therefore, the sequences between 27,607 and 30,409 were not required for proper fiber transcription. However, sequences between 27,240 and 27,608 effected fiber transcription. A polyadenylation signal for L4 is present in this region [14] and the 6.0 kb transcript found with the E3(X) deletion may represent L4 transcript(s) using the fiber polyadenylation signal, even though the E3 polyadenylation signal was intact in E3(X). The change in relative amount of fiber mRNA could be reflective of the differential regulation of polyadenylation signal usage by the late transcription unit.

Despite the altered late gene expression, the rAd35(d8) vector with the E3(X) deletion was efficiently rescued and grew to high titers. However, the productivity of the rAd35(d8) with the E3(X) deletion was 2- to 3-fold lower than the E1-deleted rAd35(d8) (data not shown). The effects of the E3 deletions were further evaluated. The relative fitness of E1-deleted vectors with and without E3 deletions was determined in virus competition experiments by co-infecting 293-ORF6 cells with equal amounts of purified virus particles from two viruses and serial passaging the resultant cell-virus lysates three times. The presence of each viral genome was determined by restriction digest of total DNA harvested from each lysate. The E3(X) virus could not be detected in the lysate from the first infection (Figure 7C), whereas the E3(HE)-deleted vector genome persisted at its relative input level through the rounds of infections (Figure 7D). Therefore, the E3(X) deletion conferred a dramatic reduction in fitness relative to E1-deleted rAd35. In contrast, the double-deletion rAd35 vector E1(d8) with the largest E3 deletion, HE, showed no reduction in relative fitness and its viral productivity was comparable to rAd35 with only an E1(d8) deletion, 86,000 and 98,000 pu/cell respectively. Thus, the E3(HE) deletion maintained proper fiber transcription, viral fitness, and good virus productivity. The E3(HE) deletion provides 2801 bp of space for foreign DNA.

### Generation of triple-deletion rAd35 vectors

Identification of an appropriate E4 deletion for a triple-deletion rAd35 vector was undertaken. The two preferential E4 deletions were initially incorporated into E1-deleted rAd35. Vectors with the combination of the E1 d8 deletion with the E4(AN) and E4(dORF3-6) deletions yielded viral progeny on 293-ORF6 cells comparable to the E1-deleted vector (Table 3). Thus, multiple-deletion rAd35 vectors were generated with favorable growth characteristics, deficits in E1 and E4 function, and complete absence of homology between the virus E4 region and E4Orf6 sequences in the production cell line.

**Table 3:** Viral progeny yields of Ad35 vectors on 293-ORF6 cells

**rAd35 vector**	**Yield (pu/cell)**
	
Ad35 wt	125,893
Ad35E1(d8)	77,839
Ad35E1(d8)E4(AN)	58,875
Ad35E1(d8)E3(HE)E4(AN	36,000

To further expand the utility of rAd35 vectors a triple-deleted vector was designed. The replication-deficient vector incorporated the deletions that had the least impact on virus function while maximizing the amount of space available for heterologous sequences. The combination of E1(d8), E3(HE), and E4(AN) provided these characteristics. In addition, rAd35 vectors with this combination of deletions yielded 36,000 pu/cell, which is comparable to the E1-deleted rAd35 (Table 3). The vector has the capacity to accommodate 6,347 bps of heterologous sequence and still remain at only 100% of wild type genome size. The vector genome was stable; no rearrangements were detected by the PCR assay in the E1, E3 and E4 regions after 10 serial passages of cell-virus lysate. In addition, protein composition of the capsid was found to be indistinguishable from wild type Ad35 in SDS-PAGE analysis (data not shown). This ease of construction, genetic stability, high productivity, reduced potential to generate a replication competent adenovirus (RCA) by homologous recombination with the production cell line, and expanded heterologous packaging capacity makes the triple-deleted vector configuration particularly suitable for use with large expression cassettes and commercial manufacturing purposes. To our knowledge this is the first E1-, E3-, E4-deleted rAd35 vector produced.

## Discussion

A combination of biochemical and biological approaches was used to derive multiple rAd35 vectors with the best replication capacity and ability to accept large insertions of DNA. Analysis of the regions of the genome targeted for deletion provided information for optimal vector design for rAd35 vectors with one, two, or three deletions. The construction of rAd35 viruses with E1 and/or E4 deletions and the analyses of the E1 and E4 regions were dependent on the 293-ORF6 cell line which provided E1 and E4 complementing activity. The 293-ORF6 cell line, which expresses Ad5 E1 and Ad5 E4 ORF6 gene products, has been shown to complement replication-deficient vectors derived from many serotypes [15-17].

Transcript and cDNA clone analysis provided for the identification of splice junctions and polyA sites for E1B, pIX, and fiber transcripts, as well as the major E4 transcripts. Previously, the pIX transcript was identified to have an intron [9]. Here we identified the splice junctions and show the intron is common to E1B transcripts. This common intron had not been previously reported and may be a feature of adenoviruses that do not have a discrete promoter for pIX positioned between the E1B ORF and the pIX ORF as found in Ad5 [10]. Transcript analysis of fiber gene expression revealed that deletion of non-essential E3 region sequence can effect fiber late gene expression. Deletion of the L4 polyA signal sequence [14] in the E3 region created a large new transcript that was generated from the fiber polyA signal, thus the E3 polyA signal was skipped, at least during the late phase of infection, and fiber transcription was increased, relative to wild type Ad35 levels. This demonstrates that disruption of gene expression regulation via deletion of non-essential sequences can result in viable but under-performing rAd vectors. Previously described E3-deleted Ad35 vectors removed the putative L4 polyA signal, however, no assessment of viral productivity or stability was provided [6].

The packaging capacity of adenovirus type 5 has been reported as 105% of the wild type Ad5 genome size [13]. The resultant preparations of Ad5 with genomes of 105% were mixed populations of full length and genomes with spontaneous deletions, thus the large genome could be packaged into the Ad5 virion but there was a strong selection for shorter genomes. In contrast, the Ad35 packaging capacity reported here was defined by maintenance of genetic stability. Recombinant Ad35 vectors with wild type levels of pIX transcription could accommodate genomes up to 103.6% of wild type genome size without signs of deletions or other genetic instability.

A deletion of the E4 region provides advantages for rAd vectors. Ad35 E4 sequences were essential for replication and their removal provided a second block to replication beyond that obtained by deleting E1. Removal of the E4 region further diminishes the likelihood that the vaccine vector could revert to replication competency in the human host while simultaneously avoiding transformation potential [18]. E1- E3-, E4-deleted rAd5 vectors display marked decreases in expression of other adenovirus and cellular genes, relative to first-generation vectors possessing only the E1 or E1/E3-deletion(s) [19,20]. This property of the vector results in reduced toxicity to transduced cells both in vitro and in vivo [21] and may reduce the induction of adenovirus-specific T cell activation following immunization [19]. The E1-E3-E4- rAd35 vector would be predicted to also have similar properties. Lastly, the E4 deletion provides an additional 1075 bp of space in the vector genome for insertion of foreign DNA. Thus, the triple-deletion backbone can stably accommodate 7,600 bp of foreign DNA, based on a genome size of 103.6%.

## Conclusions

Ad35 was identified as an attractive candidate for gene transfer vectors because of hematopoietic stem cell tropism [7] and neutralizing antibodies to Ad35 are relatively uncommon in human populations. The frequency of individuals with pre-existing immunity to Ad35 was found to range from <5% in the USA and Europe to up to 20% in Africa [1,3,4]. Ad35 vectors have been extensively tested in animal models of vaccination, as well as in ongoing Phase I trials for HIV (ClinicalTrials.gov: NCT00479999, NCT00472719, NCT00801697), malaria (ClinicalTrials.gov: NCT01018459, NCT00371189), and tuberculosis (ClinicalTrials.gov: NCT01017536, NCT01198366). However, results from the preclinical studies of rAd35 vaccine vectors indicated that transgene-specific immune responses were markedly lower compared to Ad5 vectors [22-24]. The lower immunogenicity of rAd35 vectors may have benefits for gene therapy applications because administration of rAd35 vectors to the eye gave prolonged transgene expression relative to Ad5 vectors [17]. In summary, the data reported here expanded the knowledge of adenovirus 35 and vector design and will guide the derivation of complex adenovirus vectors from other serotypes.

## Methods

### Reverse-phase high-performance liquid chromatography (rp-HPLC)

Separation of proteins was accomplished on a 2 × 50 mm Jupiter 300Å C4 column (Phenomenex, Torrance, California, USA) at 0.2 ml per minute, elution by a gradient from 20 to 90% of acetonitrile in water, and trifluoroacetic acid at 0.1% was included throughout. Analytical and semi-preparative scale rp-HPLC was performed with an 1100 series Agilent LC system. Injection volumes varied between 5 μL with a maximum of 40 μL depending on viral particle concentrations. Ultraviolet absorbance at 210 nm was collected continuously throughout the HPLC analysis. The absorbance at 210 nm was normalized to absorbance at 360 nm to eliminate anomalous absorbance due to scatter. The fractions corresponding to peaks in the UV absorbance traces were frozen on dry ice for a minimum of a half-hour. The fractions were then lyophilized in a rotary lyophilizer. Using the procedures applied here minor structural proteins such as IVa2 or protease were either not observed or identifiable.

### Whole protein mass spectroscopy by MALDI-TOF

The residue in the rp-HPLC fraction tubes that remained after lyophilization was re-suspended in 50% acetonitrile and 0.1% TFA typically in a volume of less than 10% of the original collected volume. A small volume of the sample was mixed with 20 mg/ml sinnapinic acid at a 1-to-1 ratio and 2 μL of this mixture was spotted and dried on a MALDI target plate along with protein molecular weight standards such as equine myoglobin and enolase. A dedicated target plate was used that had been optimized in the mass spectrometer to correct for slight deviations in the flatness of the plate. The machine was run in linear mode and delayed extraction of ca. 50-600 ns and acceleration voltages as required for maximum signal-to-noise and resolution was utilized. The fractioned proteins were also analyzed by SDS-PAGE allowing for co-identification of the individual proteins on both SDS-PAGE and HPLC.

### Tryptic peptide mass spectroscopy fingerprints by MALDI-TOF

The dried rp-HPLC fractions were re-suspended in 50 μL of 25 mM ammonium carbonate and incubated with 0.1 μg of TPCK-treated trypsin at 37°C for 2 hours. The peptide mixture was mixed 1-to-1 with 10 mg/ml of α-cyano-4-hydroxycinnamic acid in 50% acetonitrile and 0.1% TFA in water, spotted onto a MALDI target plate with peptide standards (Bradykinin and Angiotensin) and analyzed for a peptide fingerprint following calibration. Typically, the machine was run in reflector mode and utilized a delayed extraction of ca. 50-200 ns and acceleration voltages as required for maximum signal-to-noise and resolution. Monoisotopic masses from the MALDI-TOF machine were matched to proteins by Aldente (version 18/10/2004.) Searches were generally limited to include only those proteins isolated from viral sources but not to any specific virus family. The SDS-PAGE, rp-HPLC, and mass spectrophotometry analyses determined that purified stocks of wild type Ad35 contained intact and fragmented protein II (hexon).

### Ad35 virus description

A lysate of Ad35 was obtained from the American Type Culture Collection (ATCC, catalog # VR-718D) and a viral genome cloned into a plasmid by homologous recombination [25]. The wild type virus and recombinant derivatives were generated from the cloned genome on 293-ORF6 cells when liberated from the plasmid backbone. Sequencing of the entire viral genome revealed it to be nearly identical with the published Holden sequence found at GenBank accession number AY128640. All genome coordinates referenced in this manuscript are therefore based on the Holden sequence. Viral genomes were modified by homologous recombination in bacteria essentially as previously described [25,26]. The CMV immediate early promoter and SV40 early polyadenylation signal expression cassette in the E1 region [27] is oriented to direct transcription toward the left ITR of the virus except for those viruses with the d2 deletion in which case it is in the opposite direction. The junctions of the Ad35 deletions are indicated in the text. The P5 promoter which directs pIX transcription in the Ad35E1(d5) configuration correspond to AAV bp 143-309 (ATCC accession number AF043303). The fiber shaft modified virus (Figure 1B) is an E1(d2) configuration with a bovine growth hormone polyadenylation signal and expressing HIV glycoprotein 140B in which Ad35 sequences 31,222-31,797 are replaced with Ad5 sequences 32,239-32,787 (GenBank accession number M73260.1) was compared to wild type virus.

### Viral vector growth competitions

Virus with either E3 wild type (wt) or E3 deletions (X, HE) were competed pair wise against each other. All of the viruses contained a GFP expression cassette in the E1 (d8) deletion and are therefore E1-deleted or E1-, E3-deleted. Equal number of virus particles (pu) were mixed and 293-ORF6 cells were infected at 200 pu/cell total. 72 hours post infection cells were harvested and freeze thawed 3 times. Subsequently three sequential infections were carried out using 50 ul of crude lysate to infect a 60 cm plate of 293-ORF6 cells in a total volume of 500 ul before an additional 3.5 ml of DMEM with 10% FBS and 10 mM Zn Cl_2 _was added. The infections lasted 72 hrs. Relative levels of viral genomes were determined following the isolation of total DNA from the mixed input viral particles of a 200 μl aliquot of lysate using the High Pure Nucleic Acid Kit from Roche Applied Science (catalog # 11858874001). To distinguish the E3 wild type and E3 deletion X and wild type from deletion HE, the viral genomes were restricted with EcoRV and BlpI endonucleases (New England BioLabs), respectively, before being resolved on a 0.8% agarose gel using 0.5% TBE buffer.

### Virion temperature lability

Wild type Ad35, an rAd35 with the E1 deletion d2 and GFP expression cassette, or an rAd35 with the E1 deletion d5 and HIV gp140BΔCFIΔV1V2 expression cassette were diluted in triplicate to 8 × 10^10 ^particles/ml in 10 mM Tris pH7.5, 150 mM NaCl to a final volume of 800 ul in 1.5 ml microcentrifuge tubes on ice, then shifted to 48°C in a water bath. A 150 ul aliquot was removed at each time point, mixed with ice-cold 150 ul of DMEM + 5% FBS and kept on ice until tested for activity in the focus forming unit (FFU) assay. The data were presented as percentage of maximum activity because the input concentrations of the viruses were standardized to particle units and the three stocks of viruses had a 50-fold range in pu:FFU ratios.

### Lambda cDNA library construction and cDNA sequencing

To construct the cDNA library total RNA was isolated using the PerfectPure RNA Cultured Cell Kit (catalog # 230240) from 5Prime using the human lung carcinoma cell line A549 24 hours post infection with Ad35 wild type at a moi of 5 FFU/cell. A lambda cDNA library was constructed by Lofstrand Labs Limited (USA) with a ZAP Express cDNA Synthesis Kit from Stratagene (catalog # 200403). Phage manipulation and screening were done essentially according to *Current Protocols in Molecular Biology*.

The sequence of the pIX and fiber cDNAs were determined. Phage containing pIX and fiber cDNA sequences were identified by probing plaque lifts using DNA labeled with the North2South Biotin Random Prime Labeling kit (catalog # 17075) from Thermo Scientific. The pIX and fiber DNA probes were generated by polymerase chain reaction (PCR) with primer pairs A35s3511 (GGAGTCTTCAGCCCTTATCTGACA) + A35a3853 (CGGCCACCTGCTGAGAAA) and A35s30987 (AACAACCACAGGCGGATCTCT) + A35a31619 (CTATCATAACTAGTCATGTAGTAACATATTCCATGAATGTAGTTTTC) respectively. The identified plaques were picked and phage expanded in culture. The phage lysates were used to prime PCRs whose products were gel purified and sequenced on an ABI 3100. The entire cloned cDNA was amplified using T3 (ATTAACCCTCACTAAAGGGA) and T7 (TAATACGACTCACTATAGG) flanking primers. In addition PCR products for the 5' and 3' portions of the cDNAs were also made and sequenced. The 5' specific reactions for pIX and fiber used A35a3853 and A35a31619 respectively along with the flanking primer CCGCGGATCCGCCCAGTACATGACCTTA. Similarly the cDNA 3' specific reactions used A35s3511 and A35s30987 in conjunction with the T7 primer for pIX and fiber, respectively.

### Northern blot analysis

To generate viral RNA 293-ORF6 cells were infected at a moi of 5 FFU/cell when analyzing E1, pIX and fiber transcripts, and at a moi of 1 FFU/cell when analyzing E4 transcripts. Total RNA (5 ug) from viral and mock infected cells was harvested at 24 hours post infection except where noted in the manuscript, purified with the Versagene RNA cell Kit (Gentra Systems, catalog # VGR-0050CD), resolved on a 1 or 1.3% agarose gel containing 5% formaldehyde in 1 × MOPS buffer. The RNA was transferred to Immobilon-Ny+ (Millipore, catalog # INYC13750) rinsed sequentially in 6× SSC and water, air dried before being UV cross linked (UV Stratalinker 2400). The membrane was wetted with 5× SSPE and then prehybridized (50% Formamide, 2× SSC, 1% SDS, 5× Denhardt's and 0.5 mg/ml denatured salmon sperm DNA) at 42°C for 2-3 hours. Hybridization used the same buffer with 10% dextran sulphate overnight at 42°C along with the desired probe (see below) except for the strand specific probes which used the ULTRAhyb buffer (Ambion, catalog # AM8670). The membrane was washed sequentially twice briefly with 2× SSC, twice with 2× SSC, 0.1% SDS for 30 minutes at room temperature and three times with a solution of 0.5% SSC, 1% SDS at 56°C for 30 minutes each. The blot was air dried before being visualized using a Typhoon 9400 (Amersham Biosiences).

All probes were labeled with Redivue α-32P dCTP (Amersham, catalog # AA0075) using the Rediprime II Random Primer kit (Amersham, catalog # RPN1633) except for the strand specific probes which were DNA oligos labeled with γ-ATP using T4 kinase (New England BioLabs, catalog # M0201). The probes were: The description of the probes is as follows. The E1b and pIX probes 1-4 (Figure 3) were generated by PCR with primer pairs AGGAAGACTAGGCAACTGT + AAAGTAAGAAAAGCCACAG (probe 1), GGTGGTAATAGATACTCA + GCGTTGATGGGAAACAATATGCACAGTAGC (probe 2), CAAGCATGCCAGGTTCC + TGCGGGCAATAACCAAATGAT (probe 3), A35s3511 + A35a3853 (probe 4). The probe for the 2^nd ^intron was generated from two oligos AGTATTGGGAAAACTTTGGGGTGGGATTTTCAGATGGACAGATTGAGTAAAAATTTGTTTTTTCTGTCTTG + CAAGACAGAAAAAACAAATTTTTACTCAATCTGTCCATCTGAAAATCCCACCCCAAAGTTTTCCCAATAC. The fiber probe was generated by PCR with primer pair A35s30987 + A35a31619. The E4 probes generated by PCR used primer pairs ATAGTAGCCTGACGAACAGGT + GCTGATGAGGCTTTGTATGTG (E4 Orf1), GACAAAATATCTTGCTCCTGT + GCAGACTAATTCAAAACTAAC (E4 Orf2), ACGTGGTAACTGGGCTCTGGT + TGCTTGAAACGCAATCCAGTT (E4 Orf4).

CACCTTCCCATTTGACAGAATA + TTGGGTTTGGCGGGATT (E4 Orf6). The E4 Orf3 probe is a subcloned DNA fragment comprising Ad35 base pairs 33258 through 33611. The E4Orf6 probes were an NruI to DraIII DNA restriction fragment corresponding to Ad35 coordinates 32,008 through 32,883. The strand specific probes are A35a3853, A35a31619 and ACAACGTGGTAACTGGGCTCTGGTGTAAGGGTGATGTCTGGCGCATGATG for pIX, fiber and E4, respectively.

### Virus growth analysis

To determine the onset of viral replication, A549 cells were infected with wild type virus with 5 FFU/cell in triplicate using 60 cm plates. At the times indicated cells were washed 3× in PBS and total DNA isolated using the DNeasy Blood and Tissue kit from Qiagen (catalog # 69506). Viral genomes were quantified in a qPCR on an ABI 7700 using manufacture recommended conditions with primers CCCCGAATGGAAGTGCC and CGCTCTTTGGCGGCG along with probe 6FAMTGTTGAGTTGGACATACTCGCGTGCCMGBNFQ purchased from Applied Biosystems. The level of viral genome was normalized to ng of total DNA used in the reaction. The standard curve was generated with a plasmid of the complete E1-deleted Ad35 viral genome.

E4 complementation assays were carried out on PC-3 cells (Human prostate adenocarcinoma) infected with E1-wild type, E4-deleted viruses with 3 FFU/cell and harvested 72 hours post infection. Cells were freeze thawed 3 times and levels of active viral progeny were quantified by an FFU assay [15].

Generation of viral progeny was determined on 293-ORF6 cells which were infected with 1 FFU/cell. Cells were cultured in DMEM + 5%FBS + 100 um ZnCl_2 _and harvested 72 hours post infection. To quantify the number of particles cells were subjected to three freeze-thaw cycles, clarified through a 0.2 μm filter, and 100 ul volume was loaded onto a Poros 50D column (2 mm diameter, 10 cm length) equilibrated in 25 mM Tris pH 7.5. The column was washed with 25 mM Tris pH 7.5, vector eluted with 250 mM NaCl, 25 mM Tris pH 7.5, and particles detected by 214 nm absorbance. The number of viral particles was quantified by generating a standard curve using purified Ad35 particles standardized to A_260 _particle units [15]. All of the viruses tested had either the GFP or enhanced GFP (eGFP) gene in the E1 expression cassette.

### Vector packaging capacity

Limits on packaging capacity were assessed by testing for genetic rearrangements using a PCR. Viral genomes were liberated from its plasmid backbone by restriction digest, treated with a mixture of phenol:chlorophorm:isoamyl alcohol (24:24;1), ethanol precipitated and dissolved in water. A 60 mM plate of 293-ORF6 cells were transfected with 4 ug of viral genome using Poylyfect (Qiagen catalog # 301105). Five days post transfection cells were harvested, freeze thawed three times with 50 ul of lysate diluted to 500 ul with DMEM used to infect 60 cm plate of 293-ORF6 cells, rocked every 15 minutes for an hour before being fed with 4.5 ml DMEM + 5%FBS + 100 um ZnCl2. The virus was continued to be expanded using the same passaging regimen except cells were harvested at 72 hour post infection. To detect viral re-arrangement genomes were purified from 200 ul of lysates using the High Pure Viral Nucleic Acid kit (Roche, catalog # 11 858 874 001) in 20 ul of which 1 ul was used in a 25 ul PCR (94C 1 min, 30 cycles 94C 30 sec. × 55C 30 sec × 72C 3.5 min, with a final 72°C 10 min extension) with primers CGTACCGTGTCAAAGTCTTC and CGAGTTCTCAATGATCGAGA using Taq polymerase and Expand High Fidelity PCR buffer (Roch Diagnostics, catalog # 1435094 and 11732641001) and 1.7 ul resolved on an agarose gel.

### Genetic stability analysis

293-ORF6 cells were transfected with the rAd35 genomic plasmids, cell - virus lysates were serial passaged 3 times (cytopathic effect appeared in the first passage), and viral DNA was extracted for a polymerase chain reaction (PCR) assay [27] using primers CGTACCGTGTCAAAGTCTTC and CGAGTTCTCAATGATCGAGA.

## Authors' contributions

DM designed and carried out molecular genetic studies on viral genome modifications, participated in design and coordination of other studies, and helped to draft the manuscript. MZ and DE designed and carried out molecular genetic studies on viral genome rescues and constructions. CR conducted the biochemical studies of virion structure. DEB and GJN participated in the conception of the study. CRK contributed to design of the study. JGDG conceived of the study, participated in its design and coordination, and helped to draft the manuscript. All authors read and approved the final manuscript.

## Declaration of competing interests

A US patent application was filed on a portion of this work.

All authors except GJN are employees of a for-profit company.

The authors declare that they have no competing interests.
